# Infections With Enterohepatic Non-*H. pylori Helicobacter* Species in X-Linked Agammaglobulinemia: Clinical Cases and Review of the Literature

**DOI:** 10.3389/fcimb.2021.807136

**Published:** 2022-02-04

**Authors:** Carolina Romo-Gonzalez, Juan Carlos Bustamante-Ogando, Marco Antonio Yamazaki-Nakashimada, Francisco Aviles-Jimenez, Francisco Otero-Mendoza, Francisco Javier Espinosa-Rosales, Sara Elva Espinosa-Padilla, Selma Cecilia Scheffler Mendoza, Carola Durán-McKinster, Maria Teresa García-Romero, Marimar Saez-de-Ocariz, Gabriela Lopez-Herrera

**Affiliations:** ^1^ Laboratory of Experimental Bacteriology, National Institute of Pediatrics, Mexico City, Mexico; ^2^ Immunodeficiency Research Unit, National Institute of Pediatrics, Mexico City, Mexico; ^3^ Clinical Immunology Service, National Institute of Pediatrics, Mexico City, Mexico; ^4^ Medical Unit in Infectious and Parasitic Diseases, High Specialty Medical Unit (UMAE) Pediatrics, National Medical Center (CMN) XXI Century, Mexican Social Security Institute (IMSS), Mexico City, Mexico; ^5^ Infectious Diseases Department, National Institute of Pediatrics, Mexico City, Mexico; ^6^ Immunology, Allergy and Pediatrics Center, Angeles Lomas Hospital, Mexico City, Mexico; ^7^ Dermatology Department, National Institute of Pediatrics, Mexico City, Mexico

**Keywords:** non-*H. pylori Helicobacters*, *H. bilis*, *H. cinaedi*, X-linked Agammaglobulinemia, cellulitis, pyoderma gangrenosum-like, recurrent infections

## Abstract

The genus *Helicobacter* is classified into two main groups according to its habitat: gastric and enterohepatic. Patients with X-linked agammaglobulinemia (XLA) appear to be associated with invasive infection with enterohepatic non-Helicobacter pylori species (NHPH), mainly *H. cinaedi* and *H. bilis*. Such infections are difficult to control and have a high potential for recurrence. The spectrum of illnesses caused by these species includes recurrent fever, bacteremia, arthritis, osteomyelitis, cellulitis, abdominal abscesses, and pyoderma gangrenosum-like ulcer. The presence of these *Helicobacters* is particularly difficult to diagnose and eradicate, as they are very fastidious bacteria and present resistance to several types of antibiotics. We report two clinical cases of XLA patients infected with H. *bilis.* These infections were chronic in these patients and could not be eradicated in one of them. We also review the cases of enterohepatic non-*Helicobacter pylori* species (NHPH) in patients with this inborn error of immunity.

## Introduction

The genus *Helicobacter* includes species classified as gastric and enterohepatic. This genus belongs to a group of microaerophilic, gram-negative spiral-shaped bacteria. Among the gastric species, *H. pylori is* the most important, and its natural host is human; however, other *Helicobacter* species, both gastric and enterohepatic, have some animals as their natural host but can infect humans. Currently, several publications have shown the importance of both human and animal infection with the so-called non-*Helicobacter pylori Helicobacter* species (NHPH). Most clinically relevant enterohepatic NHPH are *H. cinaedi* and *H. bilis*, both of which colonize the intestine, biliary tree and liver of animals and humans. However, sporadic cases of *H. fennelliae, H. canis*, *H. equorum*, *H. canadiensis* and *H. pollurum* have also been reported (Fox et al., 2000; Flahou et al., 2013; Smet & Menard, 2020).

The natural hosts of *H. bilis* are mice, cats, while those of *H. cinaedii* are hamsters, rhesus macaques and dogs. Distinguishable microbiological characteristics between these two species include: *H. cinaedi* has a unipolar flagellum and is urease negative; while *H. bilis* has bipolar flagella and is urease positive (Flahou et al., 2013). These microorganisms are poorly known because they are difficult to grow from biological samples, and consequently, molecular techniques are needed for their detection and identification.

*H. cinaedi* and *H. bilis* have been associated with cases of bacteremia and systemic diseases (Holst et al., 2005; Kikuchi et al., 2012; Kim et al., 2012; Turvey et al., 2012; Shimizu et al., 2013; De Witte et al., 2016). The presence of *H. cinaedi* and *H. bilis*, which are considered the major enterohepatic NHPH associated with human diseases such as cellulitis, sepsis, osteomyelitis, and pyoderma gangrenosum (PG)-like ulcer, has been increasingly reported, particularly in immunocompromised hosts, including patients with X-linked agammaglobulinemia (XLA). However, other species have also been mentioned, such as *H. equorum, H. fennelliae*, and *H. canadiensis* (Weir et al., 1999; Schwarze-Zander et al., 2010; Funato et al., 2011; Sharp, 2011), as well as *Flexispira rappini*, a species proposed by Bryner et al., 1986. Later, in 2000, it was reported that *F. rappini* strains represent at least 10 Helicobacter taxa, and in 2005, each taxon was considered a species of helicobacter. What was until now considered *F. rappini* was demonstrated to be *H. bilis* (Dewhirst et al., 2000; Hänninen et al., 2005; Kawamura et al., 2014).

XLA is an inborn error of immunity; patients with XLA have a severe reduction in all immunoglobulin isotypes and the number of peripheral B cells is less than 2% of non-affected controls. They suffer from recurrent bacterial infections frequently affecting the respiratory tract, particularly with encapsulated bacteria, and the treatment in these patients includes the administration of monthly intravenous or subcutaneous γ-globulin. XLA is caused by mutations in *BTK* (Bruton’s tyrosine kinase), which encodes a crucial protein for B-cell development required for B-cell receptor (BCR) activation (López-Herrera et al., 2014). Several groups have reported infections with NHPH in XLA patients. Such infections are normally difficult to control, and unfortunately, international guidelines to treat these infections are not available.

Therapy with intravenous or subcutaneous γ-immunoglobulin efficiently reduces recurrent infectious diseases in patients with XLA and improves their quality of life (Durandy et al., 2005). However, some patients manifest aggressive infections with enterohepatic NHPH species despite treatment with γ-immunoglobulin, and such infections are usually resistant to treatment with several types of antibiotics and may progress to septicemia (Inoue et al., 2020). Enterohepatic NHPH species that show these types of infections include several species that are reviewed in this manuscript.

Most infections with enterohepatic NHPH species are initially detected in the skin. They are diagnosed initially as cellulitis and in some cases progress to PG-like ulcer (Harada et al., 2017). When these infections are not cleared despite antibiotic therapy, they progress to osteomyelitis and sepsis (Simons et al., 2004; Yoda et al., 2009). These infections are usually difficult to treat, as the infections are reported to have shown clinical resistance to several types of antibiotics (Cuccherini et al., 2000). For most of these infections, when resolved, broad-spectrum antibiotics are used, and it may require several months or even years of treatment to clear the infections. Here, we report two additional cases of NHPH infections and review the previous cases of NHPH reported by other authors.

## Patients and Methods

### Clinical Cases

Case 1 and Case 2 were admitted to the National Institute of Pediatrics for their previous diagnosis of XLA and the presence of skin infections. Genetic diagnosis was performed for Case 2 and confirmed for Case 1; this case was previously reported to have an exon 11 deletion in cDNA (Lopez-Herrera et al., 2008), but it was not clear if it was due to a genomic deletion or due to a splice site mutation. In both cases, a biopsy from the infected skin was obtained to perform the bacterial identification, because no pathogenic bacteria could be isolated by culture. Both patients and their guardians gave their consent to provide the samples and participate in the studies reported here. Biopsies were also used for both hematoxylin-eosin and Warthin-Starry staining.

This study was approved by the ethics, research, and biosafety committees of the National Institute of Pediatrics, SSA, project numbers: 2017/010 and 2019/024. All procedures were conducted according to the 2013 Declaration of Helsinki.

### BTK Mutation Detection

The BTK mutation in Case 1, as previously reported, is an exon 11 deletion in cDNA. To determine whether the deletion was due to a deletion in genomic DNA (gDNA) or a splice site mutation, gDNA was extracted from total blood using a Gentra Pure Gene Blood Kit (Qiagen, Hilden, Germany), and BTK exon 11 was amplified and sequenced using the following primer pairs: 5’TGTAGCTCAAGCGTGGAACTTG3’ and 5’TCTGAGGTGTGGAACACACAAC3’. For Case 2, RNA was extracted from 3x10^6^ peripheral blood mononuclear cells using the TRIzol^®^ method (Invitrogen, Walthan, Massachusetts, USA), and first strand cDNA syntesis was performed with SuperScript^®^ III reverse transcriptase (Invitrogen) following the manufacturer’s instructions. For BTK amplification, the following oligonucleotide pairs were used: BTK-P1 (forward 5’TCGAGTCCCACCTTCCAAGTC3’ and reverse 5’GGTAGTGGCTTTTTCAAGATCTG3’), BTK-P2 (forward 5’TATGGGCTGCCAAATTTTGGAG3’ and reverse 5’TGCAGTGGAAGGTGCATTCTTG3’), BTK-P3 (forward 5’ATTACCTGGCTGAGAAGCACC3’ and reverse 5’AGCCCAAATGTCAGATTTGCTG3’), and BTK-P4 (forward 5’TGGCAGCTCGAAACTGTTTGG3’ and reverse 5’TTTCTTGGCCTTTGCTCAGAAG3’). PCRs were performed in a final volume of 25μl using Taq DNA polymerase (New England Biolabs, Ipswich, Massachusetts, USA), and the melting temperatures were 59°C for P1 and P3 and 57°C for P2 and P4, with an extension time of 60 seconds. PCR products were cleaned up with exonuclease I (Thermo Scientific, Waltham, Massachusetts, USA) and alkaline phosphatase (Promega, Madison, Wisconsin, USA) for 30 minutes at 37°C, and enzymes were inactivated at 80°C for 15 minutes. Finally, sequencing reactions were performed with BigDye v3.1^®^ (Applied Biosystems, Waltham, Massachusetts, USA) and purified with BigDye Xterminator^®^ (Applied Biosystems) following manufacturing instructions. Sequencing reactions were resolved in an ABI310 genetic analyzer (Applied Biosystems) and analyzed using 4peaks software (https://nucleobytes.com/4peaks) and the reference sequence ENST00000308731.8 from ensembl (www.ensembl.org).

### DNA Extraction From Skin Biopsy Specimens

An incisional biopsy specimen was obtained from the affected skin of each patient. In Case 1, the biopsy specimen was harvested from the lesion in the right leg, and in Case 2, the biopsy specimen was harvested from the inner right leg. The biopsy samples were homogenized, and DNA was extracted from the homogenate. A total of 500 µL of the biopsy homogenate was incubated with 600 μL of buffer (10 mM Tris-HCl, pH 8.0 + 0.1% sarcosine) + 20 μL of Proteinase K (10 mg/mL) + 20 μL lysozyme (5 mg/mL) overnight at 50°C. The next day, proteinase K was inactivated by incubating at 95°C for 5 minutes. DNA extraction was carried out using the phenol-chloroform technique; DNA concentration and purity were evaluated with a Nanodrop 2000 spectrophotometer (Thermo Fisher Scientific, Waltham, MA, USA).

### NHPH Identification

*H. cinaedi* was identified by a nested PCR containing 2.0 μl of DNA template obtained from skin biopsies, 200 μM each of deoxynucleotide triphosphates (dATP, dCTP, dGTP, and dTTP), 1.25 U of DNA polymerase, and 0.25 μM of each primer (forward 5’GGAGCTGTGAGTGTGCTG3’ and reverse 5’AAATGACCGACACGAGCTG3’) in a final volume of 25 μl. The reaction conditions were 3 min denaturation at 95°C, followed by 35 cycles of 10 s of denaturing at 98°C, 30 s of annealing at 58°C, and 35 s of extension at 68°C. A final extension was at 68°C for 7 min. The PCR yielded a 659 bp product of the *cdt* gene that served as template for the second reaction using 1.0 μl of template and the set of internal primers (forward 5’GGATTTAGGCTCTCGCTCTCGTCCGGATAT3’ and reverse 5’ATCGCCGTCCTTACTCGCGTCTCCAAATTA3’). The conditions for the reaction were the same as those for the first reaction, and the final product was a fragment of 359 bp (Oyama et al., 2012).

For *H. bilis* identification, a specific PCR was performed in a final volume of 50 µL containing 5 μL of DNA obtained from skin biopsies, 0.5 µmol/L each of primers (HEBI-F1 5’-GGAAAGGGGCTTTCAATAAAG-3’, and HEBI-R1 5’-GGCTGATCCTTTAGCGAAGG-3’), 200 µmol/L of each deoxynucleotide, 1.5 mmol/L MgCl2, and 1.25 U of GoTaq^®^ Hot Start Polymerase in 1X colorless Flexi buffer (Promega, Madison, WI, USA). The amplification condition was 1 cycle at 94°C for 1 min, 55°C for 2 min, and 72°C for 3 min, followed by 39 cycles at 94°C for 45 s, 59°C for 1 min, and 72°C for 1 min, with a final extension at 72°C for 10 min. DNA from *H. bilis* strain ATCC 51630 was used as a positive control. The amplification produced a DNA fragment of 207 bp (Matsukura et al., 2002; Segura-López et al., 2015).

### *H. bilis* Sequence Analysis

>The genomic sequences were stored in a plain text file in fasta format. For sequence alignment, the BLAST program version 2.11.0 (local alignment program) for linux was used in command line mode [Scott et al., 2004]. Alignments were made between the fasta sequences of the samples and the bases of the bacterial genome data. The MEGA X program and the MUSCLE aligner were used with the UPGMA method. To view the alignments, we used the Jalview 2.11.1.4 program [Waterhouse et al., 2009]. Images of the multiple alignments were created, where the gaps, the alignment positions every 10 bases, and a consensus graph were marked with dashes. The sequences of the PCR products showed the highest alignment hit with the reported sequence NZ_JAERIZ010000077.1 *Helicobacter bilis* strain CCUG 38895. Relationships of the evolutionary history of the taxa were inferred using the UPGMA method. The evolutionary distances were calculated using the compound maximum probability method (Tamura et al., 2004).

## Results

### Clinical Cases

It is important to describe that the skin biopsies taken were taken to perform the molecular identification of non-H. pylori species. In addition, they should mention here that whole blood samples were taken for the determination of mutations to diagnose X-linked agammaglobulinemia.

**Figure 1:**
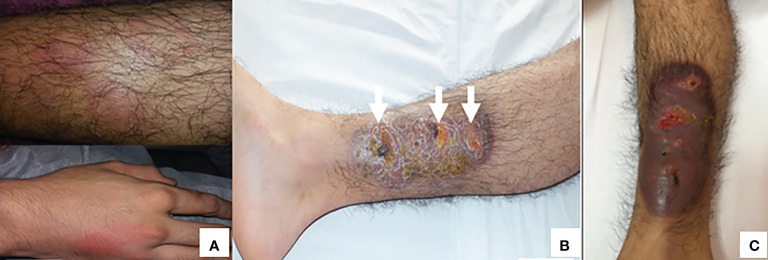
**(A)** Erythematous macules in hands and legs at the beginning of treatment (July, 2018). **(B)** The lesion in the right leg progressed to violaceous plaque and necrotic crusts (arrows) (February, 2019). **(C)** This lesions progressed, despite treatment, to pyoderma gangrenosum-like ulcer (June, 2019).

**Figure 2:**
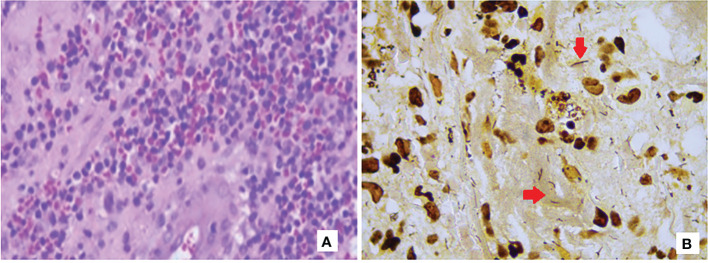
Hematoxylin-eosin **(A)** and Warthin-Starry **(B)** stainings showing a mixed inflammatory infiltrate from the skin biopsy from Case 1 and spiral-shaped bacilli among the infiltrates.

Case 1. An 18-year-old male diagnosed with X-linked agammaglobulinemia in 2008 (Lopez-Herrera et al., 2008), since then, he was on regular replacement therapy with IVIG. In March 2018 he presented mild erythematous macules in the hands and legs (**Figure 1**) accompanied by low-grade fever, followed by a painful, well-defined infiltrated 20 x 7 cm violaceous plaque (picture 2-b) with three soft fluctuating areas (white arrows) covered by necrotic crusts that partially improved with dicloxacillin and meropenem. After six months with the lesion, he came to our center, and *Helicobacter* spp. infection was suspected. Skin biopsy showed an epidermis with dense inflammatory infiltrate composed of lymphocytes and neutrophils with marked spongiosis; dermis with abundant cell infiltrate (macrophages, lymphocytes, neutrophils, and some eosinophils), evidence of vasculitis; and the Warthin-Starry stain was positive, suggesting bacillar microorganisms (**Figure 2**). PCR showed positive amplification of the *H. bilis* genetic material. An MRI showed bone edema and incipient osteomyelitis in the opposite leg, suggesting hematogenous dissemination. Due to an inflammatory infiltrate and the presence of PG-like ulcers, he received anti-inflammatory and immunosuppressive treatments (**Supplementary Figure S1**), but unfortunately, the lesion showed only mild improvement and then worsened again; we proposed hyperbaric oxygen therapy, but the patient did not consent. In March 2021, *P. aeruginosa* was isolated from the lesion surface, suggesting co-infection that was cleared after antibiotic treatment.

**Figure 3:**
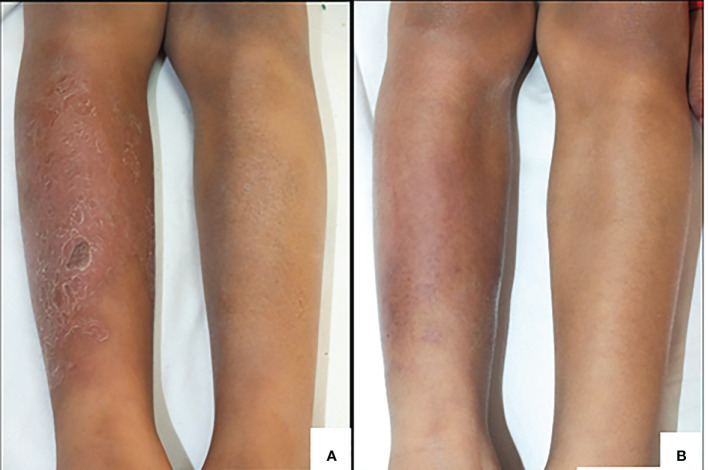
**(A)** Erythematous plaque and brownish crusts in the right leg of Case 2. **(B)** Improvement of lesion after treatment.

Case 2. A currently 11-year-old male presented with a history of cytopenia, recurrent pneumonia and sepsis, ecthyma, and hypogammaglobulinemia. He was diagnosed with X-linked agammaglobulinemia in 2014 and has received replacement therapy with human immunoglobulin regularly without severe infections since the diagnosis. The present episode started in December 2018 with a 10 x 5 cm ill-defined infiltrated erythematous plaque with scales and brownish crusts involving the right shin region extending to the lateral region of the same leg, resembling cellulitis (picture 1-a). Osteomyelitis was ruled out, but skin did not improve after treatment with anti-staphylococcal antibiotics. He was admitted to our center in January 2019, and a skin biopsy showed corneal laminar stratum with parakeratosis and orthokeratosis, some zones of hyper granulosis and psoriasiform acanthosis in the epidermis, dermis with intense cellular infiltrates (lymphocytes and histocytes), and notable lymphocytic infiltration of the subcutaneous tissue without evidence of virus, bacteria, fungi or mycobacteria. Tissue PCR identified positive genetic material for *H. bilis*. Treatment with amoxicillin plus clarithromycin, hyperbaric oxygen therapy, and local perilesional subcutaneous immunoglobulin showed clinical improvement, with lesion resolution and only residual hyperpigmentation after four weeks of treatment (**Figure 3**).

### BTK Mutations

**Figure 4:**
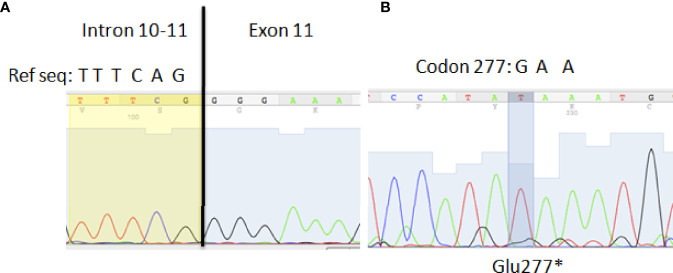
**(A)** Splice site mutation in exon 11 as the cause of the exon 11 deletion reported in Lopez-Herrera G et al., 2014. A single nucleotide deletion was identified in the 5’ splice site: TTTCAG>TTTCG. **(B)** Missense transversion (G>T) in BTK cDNA causing a premature stop codon at position 277.

Case 1 was already reported to have a deletion of exon 11 in the BTK mRNA causing a premature stop codon in residue 321 (FSdGly299*321) (Lopez-Herrera et al., 2008). To determine whether the mutation was a deletion of the region around exon 11 or a splice change, exon 11 was amplified and sequenced, and a mutation was found in the 5’ splice site of this exon (**Figure 4A**). For Case 2, the sequencing of BTK cDNA showed a missense mutation introducing a premature stop codon at position 277 (Glu277*) (**Figure 4B**).

### Presence of Enterohepatic NHPH in Biopsy of Skin

**Figure 5:**
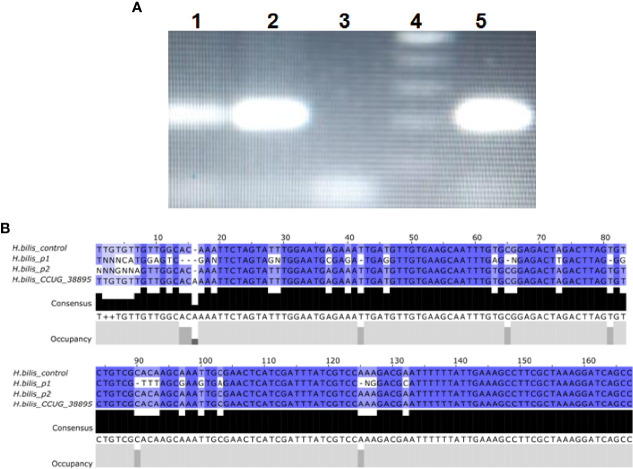
*H. bilis* identification in Cases 1 and 2. **(A)** PCR for Case 2 (1) Case 1 (2), negative control (3), molecular size marker (4), and positive control (5). **(B)** Sequence alignments of *H. bilis* 16S amplified from a control strain, Case 1, Case 2 and reference sequence from *H. bilis* CCUG 38895.

In both cases, the PCR for *H. cinaedi* was negative; however, a PCR product was amplified in the search for *H. bilis*. The 207 bp sequence derived from PCR was aligned, and analysis confirmed the presence of *H. bilis* (**Figure 5**). Comparative analysis of the 16S rRNA gene sequences of our Case 1 and Case 2 revealed an 8% sequence divergence between the two cases (p1 and p2).

**Figure 6:**
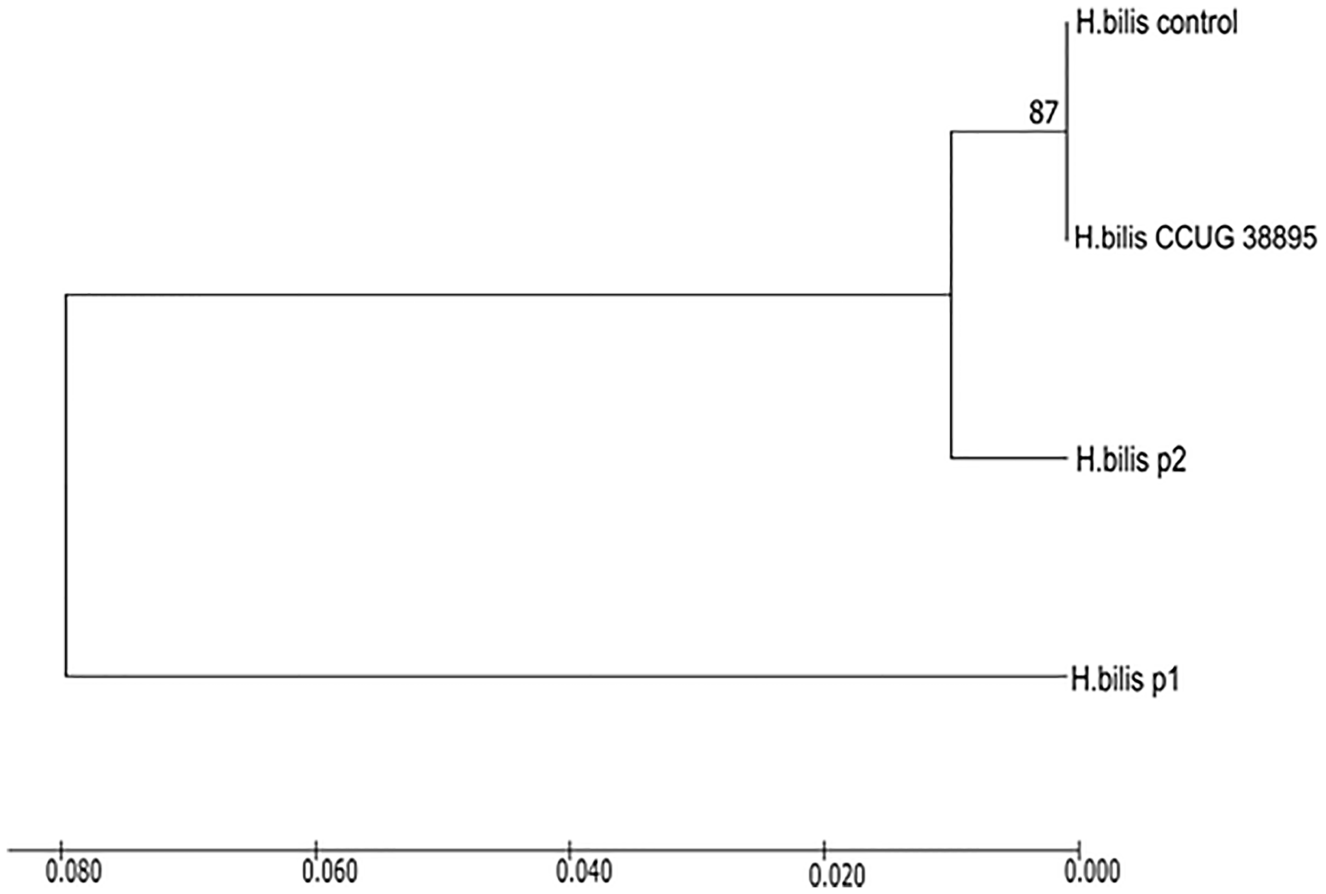
Phylogenetic tree based on partial 16S rRNA sequences showing Case 1 and Case 2 = P1 and P2, respectively, within the radiation of *H. bilis* strains. Case 2 clusters with *H. bilis* strain CCUG38895 and control strain (ATCC 51630) used in the diagnosis. The numbers at the nodes indicate the level of bootstrap support (%) based on UPGMA method analyses of 500 bootstrap replications, and percentages are indicated on nodes. The scale bar indicates an 8.0% difference in gene sequence, and distances were computed with the maximum composite likelihood method.

The phylogenetic tree was constructed to show the relationship of the sequences of both cases with *H. bilis* sequences in GenBank (**Figure 6**). A small cluster closely linked Case 2 with strains ATCC 51630 and CCUG 38895.

### Enterohepatic NHPH Infections in XLA

The literature review of patients with XLA and *Helicobacter sp* infections is summarized in **Table 1**. Of 21 cases, including the two new cases reported here, *H. cinaedi* was present in 8 cases and *H. bilis* in 8, one of which was co-infected with *H. canis*, and one case each for *H. fennelliae*, *H. equorum*, *H. canis* and *H. canadiensis*; in two cases, the species of *Helicobacter* was not identified (**Table 1**). Infections in these patients were frequently associated with cellulitis that progressed to sepsis and, in some cases, to PG-like ulcers or osteomyelitis. Cholangitis, abdominal abscess, and pleurisy was each reported in single cases (**Table 1**). Patients with these infections were treated for a few months to several years, and most of the infections were resolved; however, in two cases, the authors mentioned slow healing of PG-like ulcers. In Case 1 reported here, PG-like ulcer did not resolve after long treatment with antibiotics.

**Table 1:** Summary of case reports of NHPH infections in XLA.

Helicobacter species	Identification	Type of infection	Resolved infection	Successful antibiotic treatment	Experimental or clinical resistance to antibiotics	Reference
*Helicobacter/Flexispira-*like organism*	Cultures and 16S sequencing	4 years of worsening lower leg cellulitis	Yes	Imipenem and gentamicin.	Multiple courses of antibiotics for episodes of sepsis including ampicillin/sulbactam, trimethoprim/sulfamethoxazole, ciprofloxacin, clarithromycin, doxycycline, metronidazole, minocycline, and rifampin.	(Weir et al., 1999)
*H. bilis*	Isolation of subcultures on blood agar from blood culture bottles. *	Leg cellulitis, sepsis	Yes, after 2 years.	Imipenem, gentamicin, minocycline, metronidazole for 5 months.	Experimental resistance to trimethoprim/sulfamethoxazole, minocycline, rifampicin. nalidixic acid and cephalothin Clinical resistance to ampicillin-sulbactam, trimethoprim-sulfamethoxazole, ciprofloxacin, clarithromycin, doxycycline, metronidazole, minocycline, and rifampicin.	(Cuccherini et al., 2000)
*H.bilis*	Blood agar subcultures from blood culture bottles. *	Left knee Synovitis, skin ulcer at age 13, right leg pyoderma gangrenosum-like ulcer at age 17, osteomyelitis and sepsis at age 21	Substantial improvement of lesion.	Imipenem, gentamicin, followed by IV meropenem, 9 months	Clinical recurrence with gentamicin/metronidazole, vancomycin, nalidixic acid/cephalothin.	(Cuccherini et al., 2000)
*Helicobacter sp.*	16S sequencing of pus samples.	Left popliteal fossa tumor. Abdominal abscess on the left side of the abdominal wall.	Yes	Metronidazole and ciprofloxacin for 4 weeks.	N/S	(Han et al., 2000)
*H. canis and H. bilis coinfection*	16S sequencing of subcultures on 5% sheep blood tryptic soy, blood and chocolate agar from blood culture bottles*	Cellulitis, sepsis	Yes	Doxycycline and metronidazole for 5 months	Clinical recurrence with benzylpenicillin, amoxicillin, flucloxacillin, dicloxacillin and ceftriaxone, gentamicin, ampicillin, ciprofloxacin	(Gerrard et al., 2001)
*H. cinaedi*	16S sequencing of samples from blood culture bottles	Left forearm and foot cellulitis, osteomyelitis, sepsis	Yes	IV gentamicin and imipenem for 6 weeks	Clinical recurrence with gatifloxacin IV and oral	(Simons et al., 2004)
*H. bilis*	Mass spectrometry of isolates on blood and chocolate agar from skin biopsy*	Arm and leg cellulitis, pyoderma gangrenosum-like ulcer	Yes	IV tobramycin and meropenem for 10 weeks	Clinical recurrence with amoxicillin/clavulanate	(Murray et al., 2010)
*H. canadiensis like*	16S sequencing of samples from blood culture bottles. Subculture on 5% blood Columbia agar, 5% blood Mueller-Hinton and chocolate agar*	Leg cellulitis, sepsis	Yes, slow healing	IV amoxicillin and gentamicin changed to IV imipenem, fosfomycin for 40 weeks. Maintenance with oral rifampicin and doxycycline	Experimental resistant to cephalothin, nalidixic acid, ampicillin, azithromycin, clarithromycin, ciprofloxacin and levofloxacin. Clinical resistance to ampicillin-sulbactam, ciprofloxacin, clarithromycin, ceftazidime and clindamycin	(Schwarze-Zander et al., 2010)
*H. equorum-like*	16S and 23S sequencing of pleural samples	Refractory chronic pleurisy	Yes	Panipenem/betamipron at high doses and clarithromycin	Clinical recurrence after macrolides, cephems, newquinolones, glycopeptides and carbapenems	(Funato et al., 2011)
*H. fennelliae*	16S sequencing of bacteria on Brucella blood agar isolated from leg biopsy*	Leg pyoderma gangrenosum-like ulcer	N/S	N/S	N/S	(Sharp, 2011)
*H. cinaedi*	16S sequencing of samples from leg biopsy	Leg pyoderma gangrenosum-like ulcer, synovitis	Yes	Doxycycline and ertapenem	N/S	(Dua et al., 2012)
*H. bilis*	PCR and 16S sequencing of samples from blood culture bottles	Leg cellulitis	Yes	Ertapenem (IV), azithromycin and levofloxacin (oral) 12 months	N/S	(Turvey et al., 2012)
*H. cinaedi*	16S PCR of samples from blood culture and mass spectrometry	Leg cellulitis, sepsis	Yes	Imipenem/cilastin and minocycline	Clinical recurrence and experimental resistance to ceftriaxone/ciprofloxacin, ampicillin/sulbactam, amoxicillin/clavulanic, kanamycin	(Toyofuku et al., 2016)
*H. cinaedi*	16S PCR and mass spectrometry of samples from blood cultured bottles.	Hand and legs cellulitis, sepsis	N/S	Minocycline.	Clinical recurrence after ampicillin/sulbactam and amoxicillin.	(Sugimoto et al., 2017)
*H. bilis*	16S sequencing of samples from liver biopsy	Suppurative cholangitis	Yes	Parental ceftriaxone, metronidazole, oral doxycycline for 7 days. Oral moxifloxacin for 15 days and weekly IVIG. Azithromycin for 2 months	Clinical resistance to ofloxacin and cefpodoxime.	(Degand et al., 2017)
*H. cinaedi*	16S sequencing of samples from biopsies.	Hyperpigmented plaques in both legs and subcutaneous nodules.	Yes	Trimethoprim-sulfamethoxazole and doxycycline.	N/S	(Matsumoto et al., 2017)
*H. cinaedi*	16S sequencing of samples from blood culture bottles.	Erythematous plaques in the ankle	Yes	IV meropenem and oral doxycycline.	N/S	(Hill et al., 2018)
*H. cinaedi*	Mass spectrometry and 16S PCR of samples from blood culture bottles	Leg cellulitis, sepsis	Yes	IV imipenem/cilastin (3w), oral minocycline (12w), combined with kanamycin (15d intestinal decontamination)?	Clinical recurrence after cefazolin, ceftriaxone and oral minocycline treatment	(Inoue et al., 2020)
*H. cinaedi*	Mass spectrometry of samples from blood culture bottles.	Leg cellulitis, sepsis.	Yes	IV ceftriaxone, oral minocycline (2w), oral minocycline 18w.	Clinical resistance to IV ceftriaxone and oral amoxicillin.	(Inoue et al., 2020)
*H. bilis*	16S sequencing of samples from leg biopsy.	Hand and leg cellulitis, Leg pyoderma gangrenosum-like ulcer	After two years of antibiotics treatment and anti-inflammatory treatment, no healing of lesion was appreciated	None	Clinical recurrence after clindamycin/ceftazidime, vancomycin/meropenem, amoxicillin/clavulanate, doxycycline, cefepime, cefixime, meropenem/amikacin, sulfamethoxazole, amoxicillin/clarithromycin, metronidazole, levofloxacin, ciprofloxacin.	This report
*H. bilis*	16S sequencing of samples from leg biopsy	Leg cellulitis.	Yes	Amoxicillin/clarithromycin	N/P	This report

N/S, Not specified.

N/P, Not performed.

*Microaerophilic incubation with H_2_ and/or CO_2_ atmosphere.

Most of the species were identified by 16S sequencing or mass spectrometry of gDNA from biopsies or blood culture. Isolation on blood, chocolate or brucella blood agar in microaerophilic conditions with H_2_ and/or CO_2_ atmospheres was reported in 5 cases, but antibiotic resistance tests of the isolated bacteria were reported only in two cases. Cuccherini et al. reported resistance to trimethoprim/sulfamethoxazole, minocycline, rifampicin, nalidixic acid and cephalothin for an unspecified species of *H. bilis*, (Cuccherini et al., 2000) and Schwarze-Zander reported resistance to ampicillin, azithromycin, clarithromycin, ciprofloxacin, and levofloxacin for their isolates of *H. canadiensis* (Schwarze-Zander et al., 2010). However, most of the cases showed clinical recurrence of the infection after several types of antibiotics were used to treat patients; *H. cinaedi* and *H. bilis/F. rappini* showed clinical recurrence after treatment with quinolones, penicillins, macrolides, cephalosporins, lincosamides and aminoglycosides (Cuccherini et al., 2000; Simons et al., 2004; Murray et al., 2010; Toyofuku et al., 2016; Sugimoto et al., 2017; Inoue et al., 2020). In other cases, *H. bilis/F. rappini* also showed recurrence after treatment with carbapenems, nitroimidazoles, sulfonamides/antifolates and tetracyclines (Cuccherini et al., 2000; Degand et al., 2017). Collectively, the antibiotics reported to help in clearing the infection were broad spectrum and included a combination of two or more antibiotics and were used in the following order of frequency: carbapenems, tetracyclines, aminoglycosides, macrolides, cephalosporins, nitroimidazoles, fluoroquinolones and antifolates (**Table 1**).

## Discussion

Immunodeficient patients with NHPH infections pose challenges for treatment, particularly due to the lack of information regarding bacterial pathogenicity, antibiotic resistance, and patient factors (i.e., nutritional status, geographic factors, age) that may influence the clinical presentations of these infectious diseases. In particular, for Case 1, improvement was not achieved despite several treatments (**Supplementary Figure S1**). Both Case 1 and Case 2 were under regular and adequate immunoglobulin substitutive treatment, which suggests that the lack of antibodies alone does not completely explain the susceptibility to these microorganisms. Infections with NHPH are usually reported in the lower extremities and, less frequently, in the upper extremities, suggesting that circulatory or other local factors (trauma) may play a role in the formation or persistence of the lesions caused by colonization with NHPH. In 19 out of 21 patients (90%), the legs were involved (**Table 1**). Patients with XLA are predisposed to a pro-inflammatory state, and they are prone to develop PG or PG-like lesions (González-Serrano et al., 2012; López-Herrera et al., 2014). The growing number of reports on XLA patients with NHPH-associated skin conditions ranging from cellulitis to PG-like lesions must be a sign to alert clinicians of possible colonization by NHPH and other fastidious bacteria and consider that such infections may perpetuate the local inflammatory response. Here, inflammatory infiltrate was observed in a biopsy from a lesion of Case 1. Additionally, staining for TNF-α was positive in PG lesions from other XLA patients, but NHPHs were not reported in these patients (Schwartzfarb et al., 2008). It is not fully clear to what extent these pathogens cause direct damage to the infected tissues or whether they trigger a continuous inflammatory response in patients with immunodeficiency contributing to tissue destruction. The answer to this question may have important clinical implications, as the treatments for infection and inflammatory disease are usually based on opposing principles (antibiotics vs. immunosuppression). In our patients, we believe that both factors played a role, and therefore, both antibiotics and immunomodulatory treatments were used. The two cases reported here showed contrasting clinical responses to treatment, despite both being confirmed XLA patients with BTK truncation mutations infected with *H. bilis* (**Figure 3**). Case 2 responded favorably to treatment with antibiotics, prednisone, and hyperbaric chambers; however, in Case 1, no improvement of the lesion was observed although different schemes were used to treat with antibiotics and immunosuppressive drugs. The use of immunosuppressive drugs was suspended for a period because we considered the possibility that they were exacerbating the progression of the infection, However, the patient condition did not improve when immunosuppressive treatment was withdrawn. Of note, when the treatment with prednisone was discontinued (**Supplementary Figure S1**), the patient immediately experienced worsening of symptoms such as local pain, paresthesia and difficulty walking. In the literature, different outcomes have also been reported for these NHPH soft tissue infections.

Species from the genus *Helicobacter* constitute a potential group of emerging pathogens in various hosts, ranging from humans to animals. At present, two groups of NHPHs are recognized based on their colonization site, classified as gastric and enterohepatic. *H. cinaedi, H. bilis*, and *H. canis* are enterohepatic NHPHs that have been reported to be associated with human diseases such as bacteremia, cellulitis, cutaneous diseases, and fever of unknown origin in primary and secondary immunodeficiency. Apart from XLA, enterohepatic NHPH may also affect patients with acquired immunodeficiency syndrome (Husmann et al., 1994; Fleisch et al., 1998), patients with asplenia (Kim et al., 2012), and liver cirrhosis (Kamimura et al., 2015). In XLA, the source of the infections is unknown in most cases; however, it is suspected that they have a zoonotic origin (Owen, 1998; Schwarze-Zander et al., 2010) because many of the involved species are natural colonizers of dogs, cats, rodents, birds, pigs, sheep, and other animals (Owen, 1998). Many of the species associated with infections in humans have dogs, cats, rodents, birds, pigs, sheep, and other animals as natural hosts (Owen, 1998). Food-borne transmission has also been suggested; for example, Tee et al. reported *H. pullorum* gastroenteritis and bacteremia in immunocompetent patients who did not have previous contact with live chickens (Tee et al., 2001). Most of the cases reviewed here, including Case 1 and Case 2, showed skin infections, which are highly suggestive of zoonotic transmission after contact with infected animals; however, neither Case 1 nor Case 2 reported any contact with potential reservoirs for NHPH. Therefore, the absence of a clear epidemiological risk factor should not exclude the diagnosis in immunodeficient patients with NHPH skin infections.

Surprisingly, several reports indicate that enterohepatic NHPH species affecting XLA patients are highly resistant to treatment with several types of antibiotics, causing infection to remain for long periods of time (several years) and to affect other tissues. In contrast, in immunocompetent patients, enterohepatic NHPH infections are easy to treat. One example is the report by Kitamura T et al., in which 11 immunocompetent patients, most of whom were over 70 years old, contracted *H. cinaedi* cellulitis or bacteremia after orthopedic surgery. In most cases, the infection was cleared after completing the antibiotic treatment that included penicillins and cephalosporins with few recurrences of infections. Of note, adaptive immune response in the elderly is not as robust as in younger patients, which is an important factor that could make these patients more vulnerable to enterohepatic NHPH infections (Kitamura et al., 2007). Certainly, the immunological status of XLA patients contributes to the severity of enterohepatic NHPH infections, but it is intriguing how other antibody deficiencies, such as common variable immunodeficiency, have not been associated with NHPH infections. This raises important questions about how the patient’s vulnerability and the bacterial virulence factors interact to establish such chronic and devastating infections of enterohepatic NHPH species.

The resistance to multiple antibiotics shown by the enterohepatic NHPH species has been poorly studied. This is primarily due to the difficulty of isolating these microorganisms. Testerman et al. reported the successful growth of *H. pylori* and some NHPH species in Ham’s F-12 medium supplemented with serum and iron and incubated in specific microaerophilic conditions of CO2 and O2 (Testerman et al., 2006). However, there were no positive subcultures of *H. cinaedi* and *H. bilis*, which are the most frequent species reported in XLA and other immunodeficient patients. For this reason, it is important to focus on the culture requirements and antibiotic susceptibility of these species, which may help in the treatment of these opportunistic bacteria.

Pathogenesis mechanisms are also unknown for most enterohepatic NHPH species. Most of the patients in **Table 1**, including Cases 1 and 2 reported in this manuscript, showed skin infections started as cellulitis and progressed to other tissues. The susceptibility of XLA patients to enterohepatic NHPH infection appears to be related to the fact that XLA is associated with severe B cell (humoral) immunodeficiency; thus, these patients have difficulty with clearing intravascular or intralymphatic infection (Cuccherini et al., 2000).

Nothing is known about the bacterial mechanisms that compromise skin integrity to establish infection, colonize, stay in place and cause damage. The genomic sequence comparison between *H. pylori* and NHPH (Cao et al., 2016) as well as between gastric and enterohepatic NHPH species (Mannion et al., 2018) have shown regions into the genome unique to *H. pylori* but absent in NHPH and vice versa; a list of genes and outcomes comparing gastric and enterohepatic NHPH (shared genes and those unique to enterohepatic NHPH) may be useful in understanding mechanistic basis of its pathogenicity and adaptability.

In *H. pylori*, some of the strains that express virulence factors such as the pathogenicity island *cag*A are considered more virulent than others (Yamaoka, 2010). Similar mechanisms may be operational within the strains of *H. bilis* and *H. cinaedi* and account for differences in their ability to damage host tissues or their responses to treatment. Conversely, these strains may carry genes associated with resistance to antibiotics or types of immune responses characteristic to this group of *Helicobacters*. There are areas that need to be thoroughly investigated.

This is the first report in México on two cases of XLA and the presence of enterohepatic NHPH. *H. bilis* was the cause of chronic infections in both cases and is notoriously difficult to diagnose and eradicate. Based on the small number of publications worldwide, we highlight the typical features of *H. bilis* infection in XLA and provide a rational and successful approach to the diagnosis and treatment of this challenging infection.

In summary, clinical recommendations learned from the cases reported here and a review of the literature underscore several features that are consistently found in XLA patients infected with *H. bilis*:

Case 1 and Case 2 received different antibiotic regimens covering usual microorganisms causing cellulitis (for example, *S. aureus and S. pyogenes*), which may have played a role in generating resistance to antibiotics in NHPHs, apart from several virulence factors that a strain of *H. bilis* may carry. Given that *Helicobacter* spp. is not commonly suspected as the cause of soft tissue infections in the clinics, it is conceivable that some of the patients with PG-like ulcers may have been colonized by these or other fastidious microorganisms that perpetuate the local inflammatory response. Thus, when erythema or cellulitis is observed in XLA patients, NHPH infections should be suspected, and diagnosis using molecular techniques should be performed; otherwise, these microorganisms may not be detected. Such molecular methods are useful in identifying fastidious bacteria with special growth requirements. 16S rRNA gene sequencing should be used as the diagnostic method in XLA for timely identification of the bacteria. Additionally, we recommend performing a skin biopsy in these patients before starting treatments because histopathological findings may help to better understand the inflammatory response, to determine the existence of bacteria in the lesions, to guide some immunologically directed therapies, and to better understand the infection course and responses to treatment during the follow-up, especially in patients with poor outcomes.

The variability in treatment responses needs to be explored in more detail, as there may be factors related to microorganisms (species, virulence factors, antimicrobial resistance, etc.), to the host (nutritional status, geographic factors, age, gender, etc.), or to the health care context (delayed clinical suspicion and directed-treatment, lack of access to diagnostic methods or to broad-spectrum antibiotics, etc.). Case 2 responded well to antibiotics and hyperbaric oxygen therapy, while Case 1 did not receive the latter. There is evidence supporting beneficial effects of hyperbaric oxygen therapy in severe or refractory wounds in different scenarios, and this or other adjunctive treatments may be considered early in the course of *Helicobacter* spp. soft tissue infections. Additionally, clinicians should start treatment with antibiotics more likely to clear NHPH infections, such as carbapenems, tetracyclines, aminoglycosides, macrolides, cephalosporins, nitroimidazoles, fluoroquinolones and antifolates, and recognize that treatment may take several months. Therefore, antibiotics that may have serious side effects should be avoided.

## Conclusion

The association of enterohepatic NHPH species with XLA is becoming more solid; however, there are still certain limitations in terms of the diagnosis and treatment to eradicate this infection. Enterohepatic NHPH infection should be suspected in XLA with fever and skin lesions, and molecular methods should be employed to provide timely and adequate treatment for patients.

In immunodeficient patients with NHPH infections, clinicians face an infectious disease that is not fully understood and lacks a standardized treatment. To provide an effective treatment, further research should be performed to isolate and study the pathogenicity factors that these bacteria might possess.

## Data Availability Statement

The data presented in the study are deposited in the GenBank repository, accession numbers: OM480733, OM480734, VCV001334133.1 and VCV0013359001.1.

## Ethics Statement

The studies involving human participants were reviewed and approved by National Institute of Pediatrics Ethics Committee. Written informed consent to participate in this study was provided by the participants’ legal guardian/next of kin.

## Authors Contributions

CR-G contributed to manuscript writing, performed the identification of NHPH in biopsy samples and analyzed the data. FA-J performed the identification of NHPH in biopsy samples. JB-O, MY-N, FE-R, FO-M, SE-P, SS-M, CD-M, MG-R, and MS-d-O contributed to manuscript writing and were involved in patient treatment and follow-up. GL-H performed the genetic studies to identify BTK mutations and directed the manuscript organization. All authors contributed to the article and approved the submitted version.

## Funding

“Recursos Fiscales” from the National Institute of Pediatrics, Health Secretary, Mexico, supported this work (Project numbers: 2017/010 and 2019/024).

## Conflict of Interest

The authors declare that the research was conducted in the absence of any commercial or financial relationships that could be construed as a potential conflict of interest.

## Publisher’s Note

All claims expressed in this article are solely those of the authors and do not necessarily represent those of their affiliated organizations, or those of the publisher, the editors and the reviewers. Any product that may be evaluated in this article, or claim that may be made by its manufacturer, is not guaranteed or endorsed by the publisher.

## Supplementary Material

Supplementary Figure S1 Timeline of treatment for Case 1 in this report.
