# Supplementary figures and images for: Infections With Enterohepatic Non-H. pylori Helicobacter Species in X-Linked Agammaglobulinemia: Clinical Cases and Review of the Literature
Source: Front Cell Infect Microbiol. 2022 Feb 4;11:807136. doi: 10.3389/fcimb.2021.807136 (PMC8855360; doi:10.3389/fcimb.2021.807136)

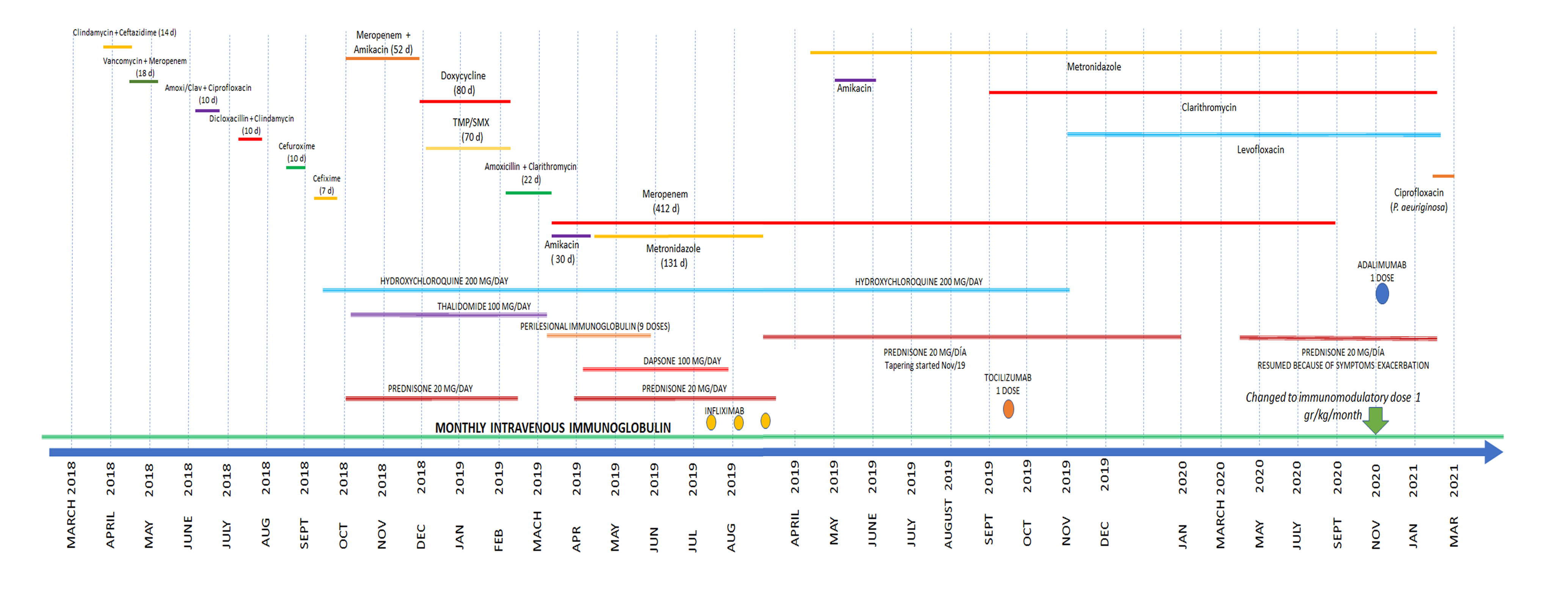

Supplement: Supplementary Figure S1 — Timeline of treatment for Case 1 in this report. [file Image_1.tif]
